# Seroprevalence of mumps before the introduction of mumps-containing vaccine in Lao PDR: results from a nationwide cross-sectional population-based survey

**DOI:** 10.1186/s13104-019-4194-3

**Published:** 2019-03-19

**Authors:** Hironori Okabayashi, Kenichi Komada, Minoru Kidokoro, Tomomi Kitamura, Shinsuke Miyano, Tomoo Ito, Kongxay Phounphenghak, Chansay Pathammavong, Keiko Murano, Misato Nagai, Yoshio Mori, Katsuhiro Komase, Anonh Xeuatvongsa, Makoto Takeda, Masahiko Hachiya

**Affiliations:** 1 Bureau of International Health Cooperation, National Center for Global Health and Medicine, Shinjuku, Tokyo Japan; 2 Department of Virology 3, National Institute of Infectious Diseases, Musashimurayama, Tokyo Japan; 3 National Immunization Program, Ministry of Health, Simuang, Vientiane, Lao PDR

**Keywords:** Seroprevalence, Mumps, Pre-vaccination era, Nationwide cross-sectional population-based survey

## Abstract

**Objective:**

Mumps-containing vaccine is currently not a component of the national immunization schedule in Lao People’s Democratic Republic (PDR). Mumps itself is not a notifiable disease in the country and the seroprevalence of anti-mumps immunoglobulin G (IgG) in the general population is unknown. In this study, anti-mumps IgG was measured in 2058 blood samples to evaluate population immunity in the country.

**Results:**

The seroprevalence of anti-mumps IgG showed a gradual increase with increasing age, starting at 10.6% (95% CI 7.4–13.7) in participants aged 1–2 years, and almost plateaued at about 75% in individuals older than 11–12 years, though it still tended toward a small increase up to 89.6% (95% CI 86.6–92.6) in participants aged 40 years or older. Compared with the results of previous studies, this increase with increasing age is less marked and the plateau of anti-mumps seroprevalence is lower. We attribute this result mainly to the lower population density in Lao PDR.

## Introduction

Mumps is a common childhood viral infectious disease that is preventable by vaccination. Live mumps vaccines are available as monovalent (mumps only), bivalent measles-mumps (MM), and trivalent measles-mumps-rubella (MMR) vaccines, and the effectiveness of at least one dose of MMR in preventing clinical mumps in children is estimated to be around 70–80% [1]. In total, 122 countries have introduced one of the mumps-containing vaccines nationwide as of 2017 [2]. Although many studies have investigated the seroprevalence of anti-mumps antibody, most recent studies were conducted after the introduction of the mumps-containing vaccine nationwide [3–6]. The majority of studies on anti-mumps antibody seroprevalence in the pre-vaccination era were conducted either in European countries before 1990 [7–10] or outside Europe [11–13]. However, the participants were not randomly selected but involved specific populations or were randomly selected but did not involve a nationwide population. To our knowledge, no studies have investigated the nationwide seroprevalence of anti-mumps antibody in a pre-vaccination era.

Lao People’s Democratic Republic (PDR) is a small developing country in Southeast Asia. It is landlocked and mountainous, and its road infrastructure is underdeveloped. The expanded program on immunization (EPI) was started in Lao PDR in 1984 but does not currently include the mumps-containing vaccine. Sporadic outbreaks of mumps have been reported, but mumps is not a notifiable disease as yet. A seroprevalence study of anti-mumps antibody has been conducted for specific populations in some areas [14], but nationwide age-specific seroprevalence in the general population is unknown.

In 2011, Lao PDR conducted a nationwide supplement immunization activity (SIA) using the measles and rubella (MR) combined vaccine targeted at children aged 9 months to 19 years. In 2014, a nationwide multistage random cluster sampling survey was conducted to evaluate the SIA by determining anti-measles and anti-rubella IgG seroprevalence among children and adults [15].

This study sought to estimate the age-specific seroprevalence of anti-mumps IgG in the nationwide general population of Lao PDR, where mumps antigen-containing vaccine is currently not included in the national immunization schedule, and analyzed the same blood samples from the 2014 survey.

## Main text

### Materials and methods

#### Study population and sampling

In this study, we used the same blood samples remaining from a nationwide multistage cluster sampling survey that we conducted in 2014 to measure anti-measles and anti-rubella IgG and evaluate the effectiveness of the SIA in 2011 [15]. The 2014 study used three-stage random cluster sampling with probability proportionate to size sampling based on the 2005 population census conducted by the Department of Statistics, Lao PDR. In total, 26 of 143 districts and 2 villages from the respective selected districts were selected, then 42 participants from each of the selected villages were selected. Participants aged 3–4 years were excluded because of indeterminate immunization history and date of birth due to inconsistencies between calendar and traditional ages that often occur in rural areas [16, 17]. From 2184 expected participants, blood samples were obtained from 2153 (97.8% of the required sample size; males 44.8%; mean age 23.2 years, age range 1–81 years). Blood sampling was performed using dried blood spots from finger prick blood spotted onto Whatman^®^ 903 Protein Saver filter paper (Whatman, Maidstone, Kent, UK) and transported to the National Institute of Infectious Diseases, Japan, within a few weeks [18, 19].

#### Anti-mumps IgG measurement

In the 2014 study, anti-measles IgG and anti-rubella IgG levels were evaluated for 2135 samples after exclusion of 18 samples due to missing vital information from 2153 samples collected between January and February. The remaining samples were stored at 4 °C as dried blood spots collected on the filter paper. Of these 2135 samples, 2058 had enough dried blood spots to measure anti-mumps IgG. Blood samples were extracted from the dried blood spots from May to July 2015 and stored at − 80 °C. Measurements were made from April to July 2017 with a commercially available enzyme-linked immunosorbent assay (ELISA) kit (Enzygnost^®^ Anti-Parotitis Virus/IgG, Siemens Healthcare Diagnostics, Eschborn, Germany) according to the manufacturer’s instructions. Anti-mumps IgG levels were determined as positive (ΔA > 0.200), equivocal (0.100 ≤ ΔA ≤ 0.200), or negative (ΔA < 0.100) according to the manufacturer’s instructions. Only the samples determined as positive were regarded as ‘positive’ for analysis.

#### Data entry and statistical analysis

All collected data were double-entered and cleaned in Microsoft Excel 2016 spreadsheet. Statistical analysis was performed and IgG seroprevalence was calculated using STATA versions 13 and 14 (Stata Corp., College Station, TX).

### Results

**Fig. 1:**
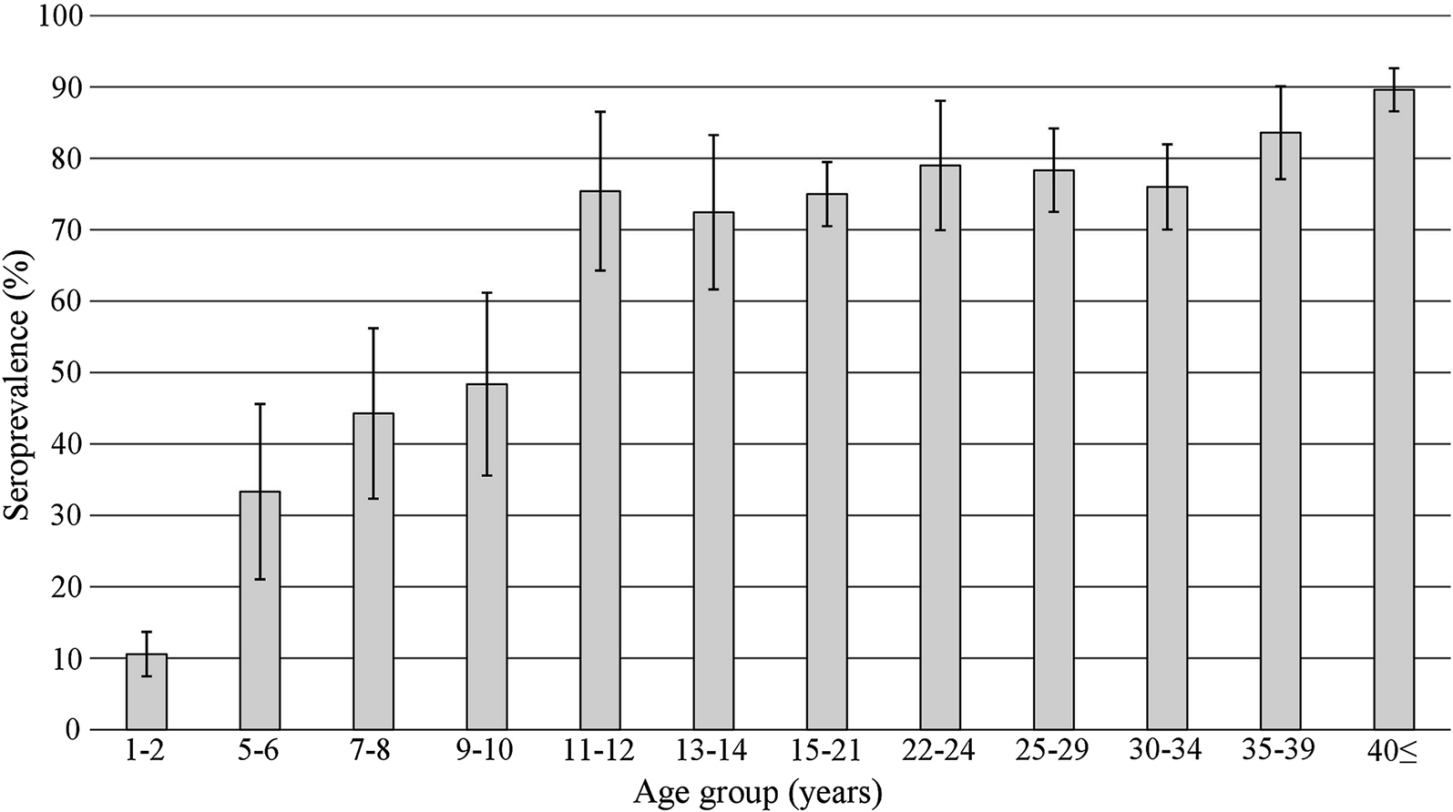
Seroprevalence of anti-mumps IgG. A total of 2058 samples were tested using a commercially available ELISA kit and a positive determination was made according to the manufacturer’s instructions. Error bars represent 95% confidence intervals

The seroprevalence of anti-mumps IgG for participants aged 1–2 years was the lowest among all age groups, at 10.6% (95% CI 7.4–13.7), and showed a gradual increase with increasing age (Fig. 1). That for participants aged 5–6 years, 7–8 years, and 9–10 years was 33.3% (95% CI 21.1–45.6), 44.3% (95% CI 32.4–56.2), and 48.4% (95% CI 35.6–61.2), respectively. Anti-mumps IgG seroprevalence reached 75.4% (95% CI 64.3–86.5) for those aged 11–12 years and almost plateaued; however, it still tended toward a small increase with increasing age in those older than 13–14 years though it fluctuated in the 70–80% range until those for aged 30–34 years. The seroprevalence of anti-mumps IgG for participants aged 35–39 years was 83.6% (95% CI 77.1–90.1) and reached 89.6% (95% CI 86.6–92.6) for those aged 40 years or older.

### Discussion

Previous studies on age-specific seroprevalence of anti-mumps IgG before the introduction of mumps-containing vaccines have been conducted in Europe [7–10] and other countries [11–13], but ours is the first nationwide study to be conducted in Lao PDR. Our study revealed a rather moderate degree of increase in IgG seroprevalence by age until age 11–12 years when it almost plateaued at about 75%. The seroprevalence tended toward a small increase after age 13–14 years and reached almost 90% in age 40 years or older. Another study in Lao PDR, which targeted elementary to high school students in four provinces, showed findings similar to our results, such as 50.0% in age group 5–9 years, 65.6% in age group 10–14 years, and 66.9% in age group 15–19 years [14]. In contrast, previous studies in other countries showed a sharp increase in IgG seroprevalence and a higher plateau, where the increase started during infancy and rose sharply to about 80% before age 10 years and then gradually increased after around age 10 to reach almost 90% before age 30. The seroprevalence tended toward a small increase fluctuating in the 90–100% range after age 30 [7, 9–13]. To suggest an explanation for our results in Lao PDR, a comparison with these earlier studies is helpful. In Wagenvoort et al*.’*s seroprevalence study of mumps immunity before introduction of mumps-containing vaccine in the Netherlands, the estimated population density of the study area was more than the national mean population density of 409 persons per km^2^ [8], and in Liu et al.’s study in China, the population density was 767 persons per km^2^ [13]. Lao PDR had a reported population density of 27 persons per km^2^ in 2015 [20], which is much lower than that in previous studies. Low population density implies less human-to-human contact and this may be the reason for the less marked increase in anti-mumps IgG seroprevalence levels and lower plateau between teens and thirties in Lao PDR. Arroyo et al.’s study among unvaccinated children in Spain revealed that rural environment, not attending school, and lack of brothers were associated with significantly lower levels of seroprevalence in the 3- to 5-year-old age group [9]. However, to our knowledge, few studies have investigated the direct relationship between anti-mumps IgG seroprevalence levels and population density before introduction of mumps-containing vaccine. Further study is necessary to investigate the direct relationship between seroprevalence levels and population density and other factors related to human-to-human contact such as school attendance and number of siblings.

The results of the present study show that many adolescents and adults are susceptible to mumps in Lao PDR, suggesting that they might be infected if a mumps outbreak were to occur. Complications of mumps among adolescents and adults are commonly seen. Orchitis occurs in about 35% of postpubertal men and oophoritis is observed in about 5% of adult women [21]. Our results provide essential information that can be utilized for planning the introduction of the MMR vaccine in the future. In addition, however, it is necessary to add mumps to the surveillance system because it is currently not a notifiable disease. Further examination of data on the incidence of mumps by age group is also needed to help estimate the burden of the disease [22].

In conclusion, this is the first study to describe the age-specific seroprevalence of anti-mumps IgG in a nationwide general population in Lao PDR and, to our knowledge, it is the first study worldwide to describe this seroprevalence in such a population before the introduction of mumps-containing vaccine into a national immunization schedule. A study to investigate age-specific seroprevalence of anti-mumps IgG offers valuable information for planning the introduction of a mumps-containing vaccine such as MMR.

## Limitations

Firstly, we designed sampling based on the 2005 population census which was rather old though it was the latest data officially available of the time. The population growth or mobility after 2005 may affect representation of the whole population of the country at the time of the survey.

Secondly, measurement of anti-mumps IgG levels were performed about three and half years after sampling. The period of the storage at 4 °C about 15 months as dried blood spots before extraction of blood samples could affect the levels of IgG. However, the previous study reported that dried blood spots stored at 4 °C up to 17 months less likely affected sensitivity and specificity for measurement of the antibody [23].

Thirdly, numerous ELISA kits are commercially available and have been used in previous studies investigating anti-mumps IgG seroprevalence [3–6, 13]. However, the cutoff value for anti-mumps IgG levels measured using these kits is not standardized. In the present study, we followed the manufacturer’s instructions to determine the IgG levels as positive, but other studies used different quantitative ELISA values with the same Enzygnost^®^ ELISA kit (Siemens Healthcare Diagnostics) [6]. Therefore, direct comparison of age-specific mumps IgG seroprevalence is not conclusive. To better assess the seroepidemiological data, cutoff values and methods for comparison among different test kits should be standardized.

## Abbreviations

CI: confidence interval
ELISA: enzyme-linked immunosorbent assay
EPI: expanded program on immunization
IgG: immunoglobulin G
MM: measles-mumps
MMR: measles-mumps-rubella
MR: measles-rubella
SIA: supplemental immunization activity
